# Use of dupilumab as a novel treatment for refractory lichen amyloidosis

**DOI:** 10.1016/j.jdcr.2024.09.006

**Published:** 2024-09-29

**Authors:** Domenica Del Pozo, Daphne Thampy, Christina W. Sun, Sylvia Hsu

**Affiliations:** Department of Dermatology, Temple University Lewis Katz School of Medicine, Philadelphia, Pennsylvania

**Keywords:** dupilumab, lichen amyloidosis, pruritus

## Introduction

Lichen amyloidosis (LA) is a chronic primary cutaneous amyloidosis of unknown etiology. LA characteristically presents as persistent hyperkeratotic skin-colored or hyperpigmented papules, usually located on the extensor surfaces. It often presents in the fifth or sixth decades of life and is often exacerbated by persistent scratching. Treatments include topical or intralesional steroids, ultraviolet light therapies, and abrasive procedures, such as dermabrasion and carbon dioxide laser, which all have inconsistent outcomes. We present a patient with lichen amyloidosis who was successfully treated with dupilumab.

## Case report

A 49-year-old Asian woman presented with a pruritic rash on the upper and lower extremities that had persisted for 20 years. The patient had been prescribed betamethasone 0.1% ointment by a dermatologist 3 years prior, which did not improve the appearance or symptoms associated with her rash. A previous biopsy of the left leg from an outside institution was consistent with LA.

**Fig 1:**
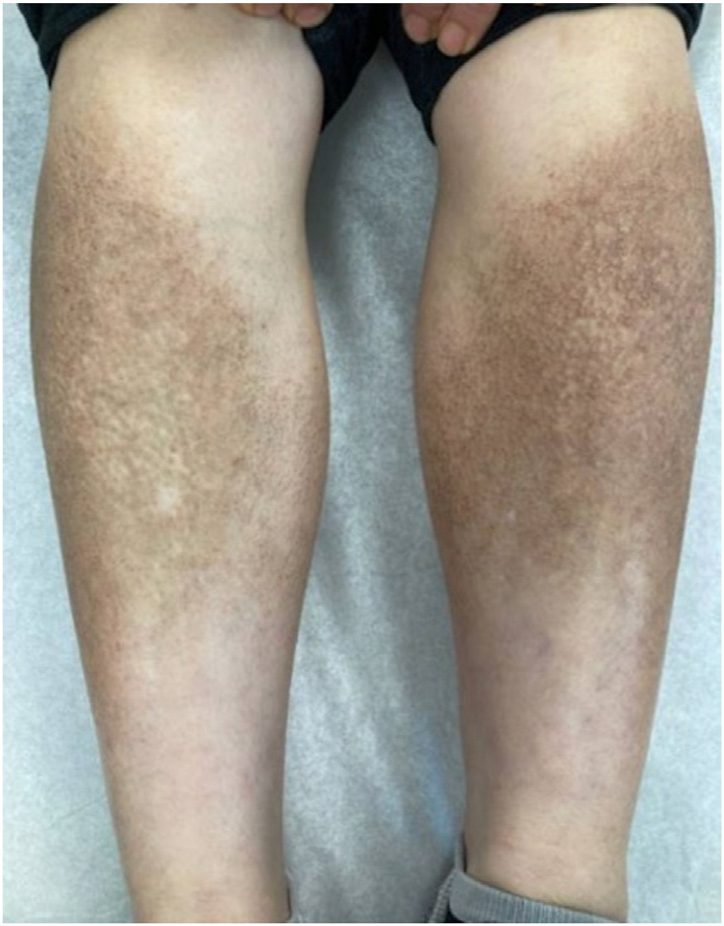
Pretreatment lichen amyloidosis. Hyperpigmented papules on the bilateral lower extremities before treatment with dupilumab.

**Fig 2:**
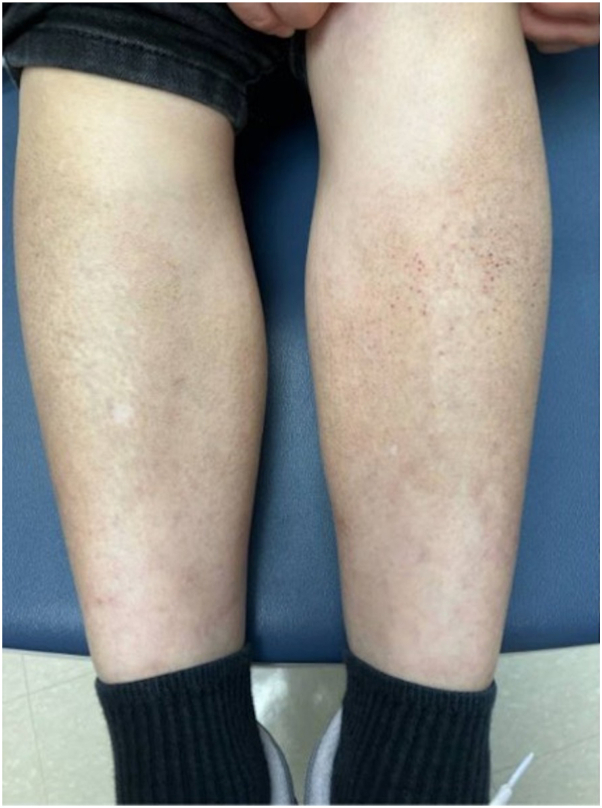
Post-treatment lichen amyloidosis after 9 months of dupilumab therapy.

Physical examination showed hyperpigmented papules on the shins and forearms (Fig 1). Initially, the patient received topical clobetasol propionate 0.05% and tacrolimus 0.1% ointments. After a month of failed topical management, the patient was started on dupilumab with a loading dose of 600 mg and a maintenance dose of 300 mg once every 2 week. Significant improvement of lesions was first noted after 3 months on dupilumab and near resolution after 9 months (Fig 2).

## Discussion

LA is one of the most common types of familial primary localized cutaneous amyloidosis (PLCA).^1^ It occurs when amyloid derived from degenerated keratinocytes deposits into the skin.^2^ Characteristically, LA presents as hyperkeratotic, hyperpigmented, pruritic papules on shins and forearms that can coalesce to form a classic rippled pattern.^3^^,^^4^ Histology typically shows amorphous, fractured, eosinophilic amyloid deposits in the papillary dermis along with hyperkeratosis and acanthosis of the epidermis.^3^

Although the pathogenesis of LA is unknown, pruritus is hypothesized to be the driving factor. Chronic pruritus leads to repeated trauma which causes damage to keratinocytes and deposition of keratin-derived amyloid within the papillary dermis.^5^ Studies have suggested that T helper 2 cells secrete interleukin (IL)-31, which may play a role in the itch pathway for PLCA.^6^

Popular treatment options for LA include topical corticosteroids, topical tacrolimus, antihistamines, ultraviolet light therapy, cyclosporine, isotretinoin, dermabrasion, and laser therapy.^4^ While current management for LA places a focus on improving associated pruritus, it often fails to resolve cutaneous changes. Dupilumab is a human monoclonal IgG antibody directed against the alpha chain of the IL-4 receptor. It inhibits the signaling of IL-4 and IL-13, which drive inflammatory skin conditions, like atopic dermatitis.^7^ Dupilumab may also block T helper 2 cells from releasing IL-31, curtailing the itch pathway associated with PLCA.^8^ The use of dupilumab for the treatment of LA has been scarcely reported in literature. This case report supports the use of dupilumab to not only relieve symptoms associated with LA, but to completely resolve its cutaneous manifestations.

## Conclusion

The mechanism of action for how dupilumab successfully treats individuals with LA is still unclear and requires further investigation. However, this report supports the use of dupilumab as a safe, effective, and novel treatment for managing LA.

## Conflicts of interest

None disclosed.
